# Forest Type Shapes Soil Microbial Carbon Metabolism: A Metagenomic Study of Subtropical Forests on Lushan Mountain

**DOI:** 10.3390/microorganisms14010220

**Published:** 2026-01-17

**Authors:** Dan Xi, Feifei Zhu, Zhaochen Zhang, Saixia Zhou, Jiaxin Zhang

**Affiliations:** 1 Jiangxi Provincial Key Laboratory of Carbon Neutrality and Ecosystem Carbon Sink, Lushan Botanical Garden, Jiangxi Province and Chinese Academy of Sciences, Jiujiang 332900, China; 2 Institute of Applied Ecology, Chinese Academy of Sciences, Shenyang 110016, China

**Keywords:** microbial functional genes, carbon fixation, vegetation type, carbon-degrading enzymes, forest ecosystem

## Abstract

Forest type strongly influences soil microbial community composition and associated carbon cycling, yet its influence on microbial functional traits remains poorly understood. In this study, metagenomics sequencing was used to investigate soil microbial communities and carbon metabolism genes across three forest types: deciduous broadleaf (DBF), mixed coniferous–broadleaf (CBMF), and coniferous forest (CF) at two soil depths (0–20 cm and 20–40 cm) on Lushan Mountain in subtropical China. The results showed that CF exhibited higher bacterial diversity and a distinct microbial composition, with an increase in Actinomycetota and Bacteroidota and a decrease in Acidobacteriota and Pseudomonadota. The Calvin cycle was the dominant carbon fixation pathway in all forests, while the relative abundance of secondary pathways (i.e., the 3-hydroxypropionate bi-cycle and reductive citrate cycle) varied significantly with forest type. Key carbon fixation genes (sucD, pckA) were more abundant in CF and CBMF, with higher levels of rpiA/B and ackA in DBF. Functional profiling further indicated that CF soils, especially in the surface layer, were enriched in glycoside hydrolases (GHs) and carbohydrate esterases (CEs), while CBMF showed a greater potential for starch and lignin degradation. Multivariate statistical analyses identified soil available phosphorus (AP) and pH as primary factors shaping microbial community variation, with AP emerging as being the dominant regulator of carbon-related functional gene abundance. Overall, the prevalence of these distinct genetic potentials across forest types underscores how vegetation composition may shape microbial functional traits, thereby influencing the stability and dynamics of the soil carbon pool in forest ecosystem.

## 1. Introduction

Forest soils, as one of the largest terrestrial carbon (C) reservoirs, play a key role in climate regulation [1,2]. Microorganisms are the primary drivers of C transformation, regulating key soil C–cyclingprocesses including organic matter decomposition, C fixation, and mineralization [3,4,5]. The functional potential of soil microbial communities, reflected in the abundance and diversity of metabolic genes, is therefore central to understanding their capacity to modulate C storage and turnover in forest ecosystems [6,7,8]. However, this complexity, driven by interacting factors such as tree species composition, soil properties, and climate, creates substantial uncertainty in scaling predictions of microbial C processes. Consequently, a critical knowledge gap persists in our mechanistic understanding of how microbial functional traits govern ecosystem-scale C dynamics.

Forest type is a major determinant of soil microbial composition, with cascading effects on soil organic C dynamics [9,10]. Changes in forest vegetation can directly alter the quantity and quality of litter inputs and root exudates, which modify nutrient availability and substrate composition, ultimately driving shifts in microbial community structure and function [11,12]. These shifts can have profound effects on C–cycling processes [13,14]. It has been reported that nutrient availability significantly regulates microbial C-fixation rates by influencing community composition [15]. Furthermore, microbial C fixation has been positively linked to tree species diversity in subtropical forests [16]. Despite growing recognition of the microbial role in forest C-ycling, considerable gaps remain at the functional genetic level [3,12]. In particular, systematic comparisons of the distribution and expression of key functional genes, such as these involved in C-fixation and degradation pathways across different forest types, are still lacking. Metagenomics enables comprehensive profiling of microbial functional genes, offering insights into the metabolic potential of both cultivated and uncultured microorganisms [17,18]. Therefore, applying functional metagenomic analyses across diverse forest types presents a promising tool to decipher the genetic mechanisms underlying microbial C metabolism and its response to vegetation-induced environmental changes.

Lushan Mountain, characterized by distinct vertical vegetation zonation and diverse soil types, offers a natural laboratory for studying biogeochemical cycles under environmental gradients [19]. Previous studies in this region have found that vegetation type and altitude are strongly linked to soil organic C and nitrogen (N) dynamics [19,20,21,22]. However, how microbial C metabolic functions respond to forest type remains largely unexplored. To address this gap, we collected soil samples from the three typical forests on this mountain: deciduous broadleaf forest, conifer–broadleaf mixed forest, and conifer forest and conducted metagenomic sequencing. Our specific study aims are as follows: (1) compare soil microbial community composition and diversity among these forest types; (2) characterize the patterns of microbial C-fixation and carbohydrate degradation functions; and (3) identify the key environmental factors regulating these C-related metabolic processes.

## 2. Materials and Methods

### 2.1. Site Description and Sample Collection

The study was carried out at Lushan National Nature Reserve (29°25′ N–29°41′ N, 115°51′ E–116°07′ E), located in Jiujiang City, Jiangxi Province, China. The region has a subtropical monsoon climate, with an average annual temperature of 11.6 °C and an average precipitation of 2068.1 mm. Soil types change with altitude, including Ferric Alisols, Haplic Alisols, Haplic Umbrisols, and Haplic Argosols [21].

Our sampling sites were set in three distinct forest types: deciduous broadleaf forest (DBF, 115°56′5″ E, 29°31′34″ N, 1018 m a.s.l.), conifer–broadleaf mixed forest (CBMF, 115°56′8″ E, 29°31′26″ N, 1106 m a.s.l.), and conifer forest (CF, 115°56′17″ E, 29°31′24″ N, 1213 m a.s.l.). The DBF was dominated by the tree species *Sorbus folgneri* and *Quercus glandulifera*, with *Rhododendron simsii* and *R. mariesi* composing the main shrub layer. In the CF, *Pinus taiwanensis* was the primary tree species, accompanied by some shrubs such as *R. simsii* and *Hypericum monogynum*. The CBMF featured a mixed canopy of *P. taiwanensis*, *Platycarya strobilacea*, and *Cornus kousa* subsp. *chinensis*. In August 2023, three 10 m × 10 m replicate plots were established in each forest type. In each plot, six soil cores (3.0 cm diameter) were randomly taken from two depth intervals (0–20 cm and 20–40 cm) after removing surface litter. Soil cores from the same depth were pooled to form one composite sample per depth per plot, yielding a total of 18 composite samples (3 forest types × 3 plots × 2 depths). Additionally, undisturbed soil samples were collected at each depth using 100 cm^3^ ring knives for bulk density (BD) determination. All samples were placed in sterile plastic bags and transported to the laboratory on ice. The composite samples were passed through a 2 mm sieve after visible roots and debris were removed. Sieved soils were divided into two parts: one part was stored at −20 °C and used for DNA extraction and metagenomic sequencing, and the other part was stored at 4 °C or air-dried for identification of the soil physicochemical properties, including pH, soil water content (SWC), total organic carbon (TOC), total nitrogen (TN), total phosphorus (TP), inorganic N (e.g., NH_4_^+^-N and NO_3_^−^-N), available phosphorus (AP), dissolved organic carbon (DOC), and dissolved organic nitrogen (DON).

### 2.2. Soil DNA Extraction, Metagenomics Sequencing, and Bioinformatic Analysis

Total genomic DNA was extracted from 0.25 g of fresh soil using the DNeasy^®^ PowerSoil Kit (MoBio Laboratories Inc., Carlsbad, CA, USA) according to the manufacturer’s protocols. The DNA concentration and purity were detected using a NanoDrop 200 spectrophotometer (A260/280 ≥ 1.6, NanoDrop Technologies, Wilmington, DE, USA) and Qubit 4.0 fluorometer (≥0.1 μg, Life Technologies, Carlsbad, CA, USA), respectively. The DNA extract quality was checked on a 1% agarose gel (fragments > 2 kb). Then, the constructed DNA library with insert sizes of ~400 bp was subjected to metagenomics sequencing using an Illumina PE150 platform (Illumina Inc. San Diego, CA, USA) at Wuhan Benagen Technology Co., Ltd., Wuhan, China. For each sample, an average of 10 Gb raw data was generated and the basic sequence information was listed in Table S1.

The raw sequences were processed to filter the adapters and low-quality reads (length < 50 bp, with ambiguous base “N” < 5 bp) using Fastp (version 0.23.2). To ensure the quality of the metagenomic data, we processed the raw reads using a 5 bp sliding window, trimming sequences with a quality score lower than Q20. Then, the Kraken2 (version 2.1.2) with standard nt database (download date: 15 October 2022) was used to taxonomically classify clean data in order to identify proteins from bacteria, archaea, eukaryotes, and viruses in the samples [5,23]. Subsequently, the clean reads were assembled into contigs using MEGAHIT (parameters: kmer_min = 21, kmer_max = 141; version 1.2.9) and the contigs < 500 bp were removed. The open reading frame (ORF) for each assembled contig was predicted using Prokka (parameters: gcode 11; version 1.1.6) and these were translated into amino acid sequences. A non-redundant gene catalog was constructed using CD-HIT (version 4.8.1) with 95% identity and 90% coverage.

Functional annotation of non-redundant genes was conducted using HMMER (version 3.3.2; https://github.com/EddyRivasLab/hmmer, accessed on 15 May 2024) against the KofamScan (version 1.3.0; https://www.genome.jp/ftp/db/kofam/, accessed on 15 May 2024) of the Kyoto Encyclopedia of Genes and Genomes (KEGG, download date: 3 May 2021; https://www.kegg.jp/kegg/, accessed on 15 May 2024) database and the carbohydrate-active enzymes (CAZyme, download date: 24 September 2021; http://www.cazy.org/, accessed on 15 May 2024) database with an e-value threshold of 1 × 10^−5^. The targeted C-fixation genes were extracted from the KEGG pathway modules and their respective KEGG orthology (KO) numbers and pathways were identified for each sample. Within the CAZyme database, the annotated genes were classified into auxiliary activities (AAs), glycoside hydrolases (GHs), glycosyl transferases (GTs), polysaccharide lyases (PLs), carbohydrate esterases (CEs), and carbohydrate-binding modules (CBMs). These CAZyme genes were further categorized manually based on various C substrates reported in previous studies [24,25,26]. To assess the abundance of these genes in each sample, we predicted gene sequences using Salmon [27] and the TPM (transcripts per kilobase per million mapped reads) was used to normalize the abundance values [8,28].

### 2.3. Determination of Soil Physicochemical Properties

The TOC and TN contents (g kg^−1^) were determined by an element analyzer (flash 2000HT, Thermo Fisher Scientific, Waltham, MA, USA). Soil pH was measured using a pH meter (PHS-3C, Shanghai Leici Instrument Co., Shanghai, China) in a 1:2.5 soil–water suspension. The SWC (%) and BD (g cm^−3^) were determined by the gravimetrical weight method by drying fresh soil at 105 °C for 24 h. Soil inorganic nitrogen contents (mg kg^−1^) were extracted by 2 M KCl and determined using the indophenol blue method with 625 nm for NH_4_^+^-N and using ultraviolet spectrophotometry colorimetry method with 220 and 275 nm for NO_3_^−^-N, respectively [29,30]. The above extract was then analyzed for total dissolved nitrogen (TDN, mg kg^−1^) using the potassium persulfate oxidation method [29]. The DON content (mg kg^−1^) was calculated by subtracting inorganic N from TDN [DON = TDN − (NH_4_^+^-N + NO_3_^−^-N)]. Soil TP (g kg^−1^) and AP (mg kg^−1^) were measured using the colorimetrical molybdate blue method after acid digestion with H_2_SO_4_-HClO_4_ and after being extracted with a 0.03 M NH_4_F–0.025 M HCl solution, respectively [29,31]. Soil DOC (mg kg^−1^) was extracted by 0.5 M K_2_SO_4_ and determined using a Shimadzu TOC analyzer (TOC-VCPH, Kyoto, Japan) [32].

### 2.4. Statistical Analysis

Two-way ANOVA was used to assess the effects of forest type and soil layer on soil microbial community and the abundance of genes related to C-fixation and degradation. One-way ANOVA followed by Tukey’s HSD post hoc test was used to determine differences in these variables, including soil properties, among forest types. Differences in microbial community structure across forest types and soil layers were examined using principal coordinate analysis (PCoA) with non-parametric multivariate statistical methods (ADONIS). Pearson’s correlation analysis was conducted to examine the relationships between soil properties, microbial community, and functional gene abundances using the R package “corrplot” (version 0.95). Distance-based redundancy analysis (db-RDA) with the R package “vegan” (version 2.7.1) was used to identify the soil variables that best explained variations in microbial community, C-fixation genes, and CAZyme genes. The Mantel test was conducted to evaluate the significance of associations between soil properties and microbial profiles, and the relative contribution of each soil variable was quantified using hierarchical partitioning via the “rdacca.hp” package (version 1.1.1) [33]. Differential CAZyme genes across forest types were identified through linear discriminant analysis effect size (LEfSe), with an LDA score threshold > 2.5 (*p* < 0.05). All statistical analyses were performed using R (version 4.4.1) and SPSS 27.0 (SPSS Inc., Chicago, IL, USA), with significance defined at *p* < 0.05. Data visualization was carried out using Origin Pro 2024 (Origin Lab Inc., Northampton, MA, USA) and RStudio (version 2024.04.2+ 764).

## 3. Results

### 3.1. Soil Physicochemical Properties

Soil physicochemical properties differed significantly among forest types (Table 1). DBF exhibited significantly lower contents of TOC, TN, TP, and DOC than CF and CBMF. In contrast, NO_3_^−^-N content increased significantly from DBF to PF, opposite to the trend in soil pH within the 0–20 cm layer. In this surface layer, NH_4_^+^-N and AP contents were significantly higher in CF than in DBF and CBMF. Most soil properties showed higher values in the 0–20 cm soil layer.

### 3.2. Microbial Community

**Figure 1:**
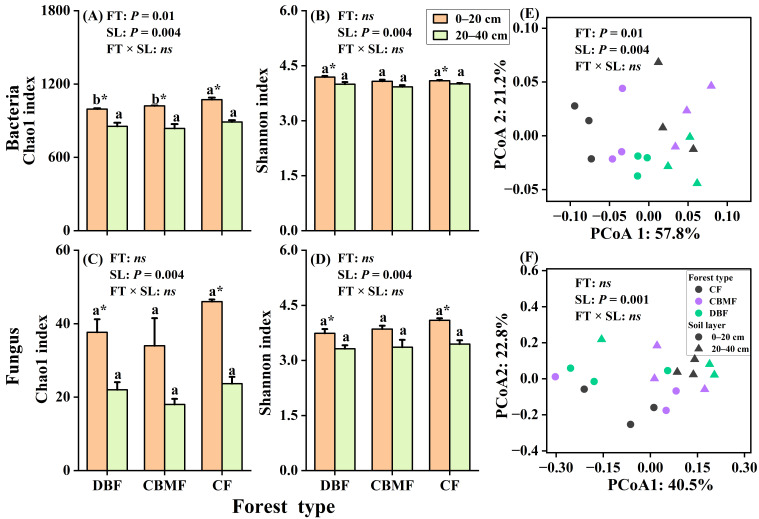
Differences in microbial diversity index (**A**–**D**) and community structure (**E**,**F**) based on principal coordinate analysis under different forest types. Different small letters indicate significant differences among forest types and the * indicates significant differences between soil layers at a 0.05 significance level. FT, forest type; SL, soil layer; FT × SL: the interaction of forest type and soil layer; *ns*, no significant difference at a 0.05 significance level. CF, conifer forest; CBMF, conifer–broadleaf mixed forest; DBF, deciduous broadleaf forest.

**Figure 2:**
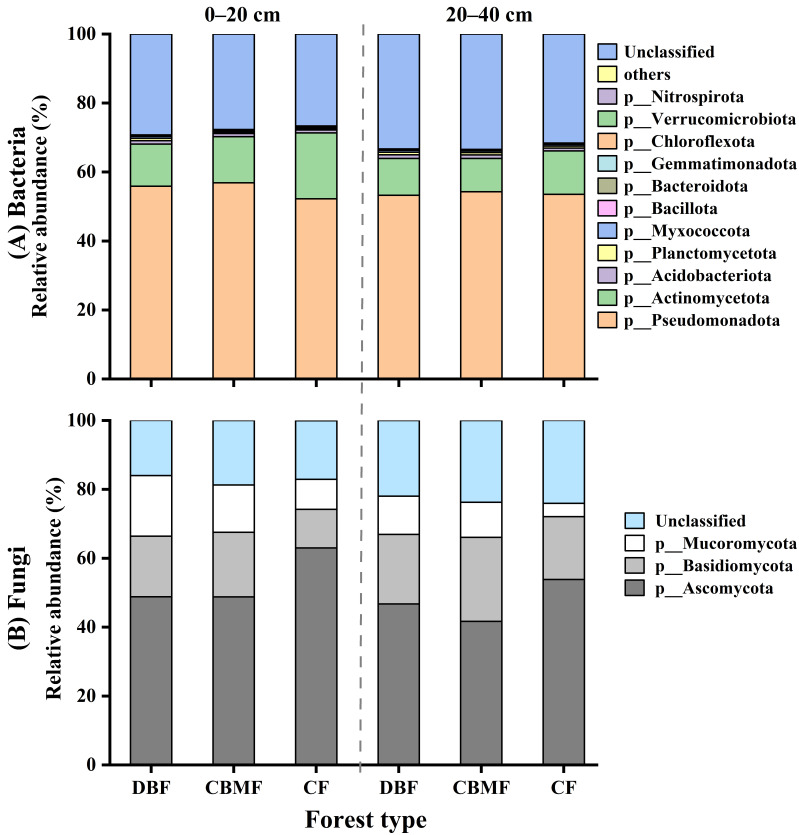
Relative abundance of soil bacterial (**A**) and fungal (**B**) communities at the phyla level in different forest types. Those phyla that represent > 0.01% of the bacterial community abundance are named, while those that represent < 0.01% of the bacterial community abundance are referred to as “others”. CF, conifer forest; CBMF, conifer–broadleaf mixed forest; DBF, deciduous broadleaf forest.

Metagenomic taxonomic analysis showed that bacteria dominated soil taxa, followed by minor proportions of fungi and archaea (Table S2). Bacterial diversity and community composition were significantly influenced by forest type, soil layer, and/or their interaction, while fungi diversity was affected only by soil layer (Figure 1, Table S3). Soil bacterial chao 1 index in the 0–20 cm layer was significantly higher in CF than in CBMF and DBF (Figure 1A). Microbial diversity in CF and DBF, as well as bacterial chao1 index in CBMF, were significantly higher in the 0–20 cm layer than 20–40 cm layer (Figure 1). At the phylum level, Pseudomonadota (52.2–56.9%) and Actinomycetota (9.7–19.1%) were the most abundant bacterial groups, while Ascomycota (41.7–63.0%), Basidiomycota (11.2–24.4%), and Mucoromycota (3.8–17.6%) predominated among fungi (Figure 2, Table S3). Compared to DBF and CBMF, CF showed higher relative abundances of Actinomycetota, Bacteroidota, and Ascomycota, but lower abundances of Pseudomonadota, Bacillota, and Acidobacteriota (Table S3). Conversely, DBF exhibited a higher abundance of Planctomycetota and Myxococcota. With increasing soil depth, the relative abundances of Actinomycetota and Bacteroidota decreased, whereas other microbial groups such as Planctomycetota and Basidiomycota increased (Table S3).

### 3.3. C- Fixation Pathways and Genes

**Figure 3:**
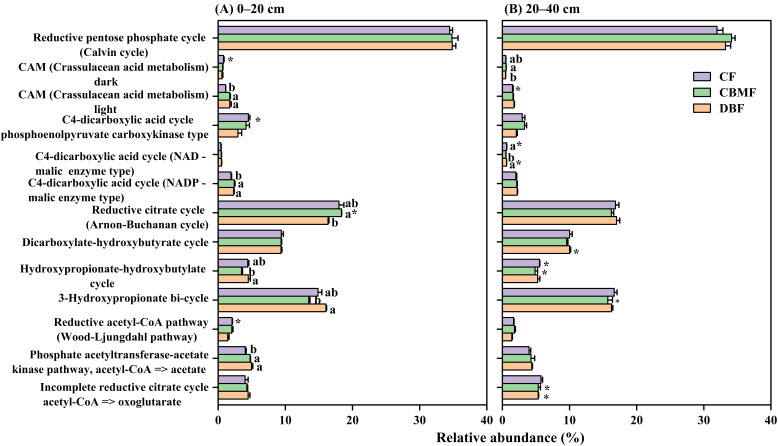
Changes in relative abundances of C- fixation pathways among forest types in the 0–20 cm (**A**) and 20–40 cm (**B**) soil layer. Different small letters indicate significant differences among forest types, and the * indicates significant differences between soil layers at a 0.05 significance level. CF, conifer forest; CBMF, conifer–broadleaf mixed forest; DBF, deciduous broadleaf forest.

Ten C-fixation pathways were detected across all forest soils within the KEGG database (Table S3). The highest and lowest relative abundances were the reductive pentose phosphate (Calvin) cycle and reductive acetyl-CoA pathway (WL pathway), respectively (Figure 3). Forest type and soil layer significantly affected the C4-dicarboxylic acid cycle, hydroxypropionate-hydroxybutylate cycle, and 3-hydroxypropionate bi-cycle. In contrast, the CAM cycle and WL pathway were influenced only by forest type, while the reductive citrate cycle and Calvin cycle were mainly affected by soil layer or its interaction with forest type (Table S4). In the 0–20 cm layer, DBF showed higher relative abundances of the 3-hydroxypropionate bi-cycle and hydroxypropionate-hydroxybutylate cycle than CBMF, whereas the opposite pattern was observed for the reductive citrate cycle, and CF exhibited lower relative abundances of the CAM (light) cycle, phosphate acetyltransferase-acetate kinase pathway, and C4-dicarboxylic acid cycle (NADP-malic enzyme type) (Figure 3A). In the 20–40 cm layer, CBMF showed a higher relative abundance of the CAM (dark) cycle and a lower abundance of the C4-dicarboxylic acid cycle (NAD-malic enzyme type) (Figure 3B).

**Figure 4:**
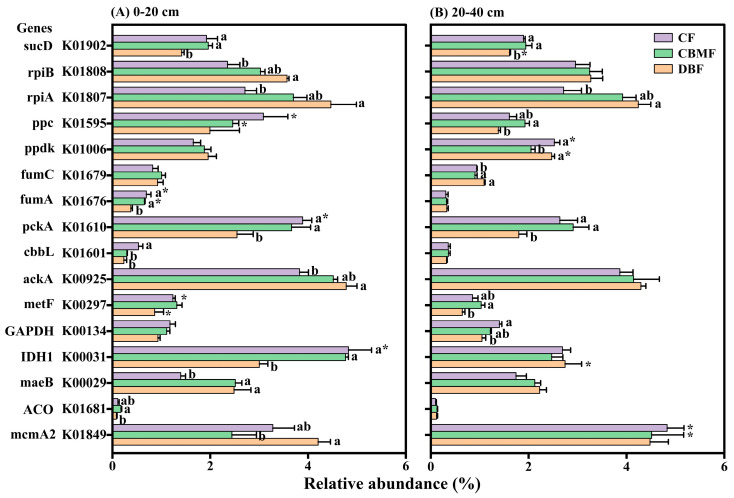
Different carbon fixation genes among forest types in the 0–20 cm (**A**) and 20–40 cm (**B**) soil layer. Different small letters indicate significant differences among forest types, and the * indicates significant differences between soil layers at a 0.05 significance level. CF, conifer forest; CBMF, conifer–broadleaf mixed forest; DBF, deciduous broadleaf forest.

At the gene level, a total of 57 genes (KO numbers) were identified across C-fixation pathways, with several (i.e., *maeB*, *sdhA/B/C*, and *korA/B*) contributing to multiple pathways (Table S4). Specifically, the relative abundances of *sucD*, *pckA*, *fumA*, and *IDH1* were significantly higher in CF and CBMF than in DBF, whereas *mcmA2*, *rpiA/B*, and *ackA* were enriched in DBF (Figure 4). The relative abundance of cbbL in the 0–20 cm layer was significantly higher in CF compared to DBF and CBMF (Figure 4A). Conversely, opposite trends were observed for *maeB* (0–20 cm) and *fumC* (20–40 cm). Moreover, CBMF exhibited higher relative abundances of *ACO* in the 0–20 cm layer and of *ppc* and *GAPDH* in the 20–40 cm layer, compared to DBF.

### 3.4. Carbohydrate Active Enzyme Genes

**Figure 5:**
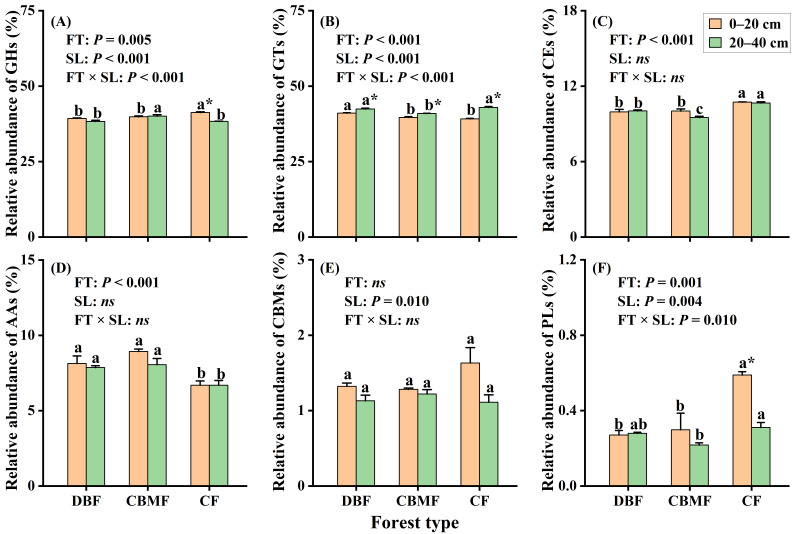
Changes in relative abundance of CAZyme families among forest types. (**A**) the relative abundance of glycoside hydrolases (GHs); (**B**) the relative abundance of glycosyl transferases (GTs); (**C**) the relative abundance of carbohydrate esterases (CEs); (**D**) the relative abundance of auxiliary activities (AAs); (**E**) the relative abundance of carbohydrate-binding modules (CBMs); (**F**) the relative abundance of polysaccharide lyases (PLs). Different small letters indicate significant differences among forest types, and the * indicates significant difference between soil layers at a 0.05 significance level. FT, forest type; SL, soil layer; FT × SL: the interaction of forest type and soil layer; *ns*, no significant difference at a 0.05 significance level. CF, conifer forest; CBMF, conifer–broadleaf mixed forest; DBF, deciduous broadleaf forest.

**Figure 6:**
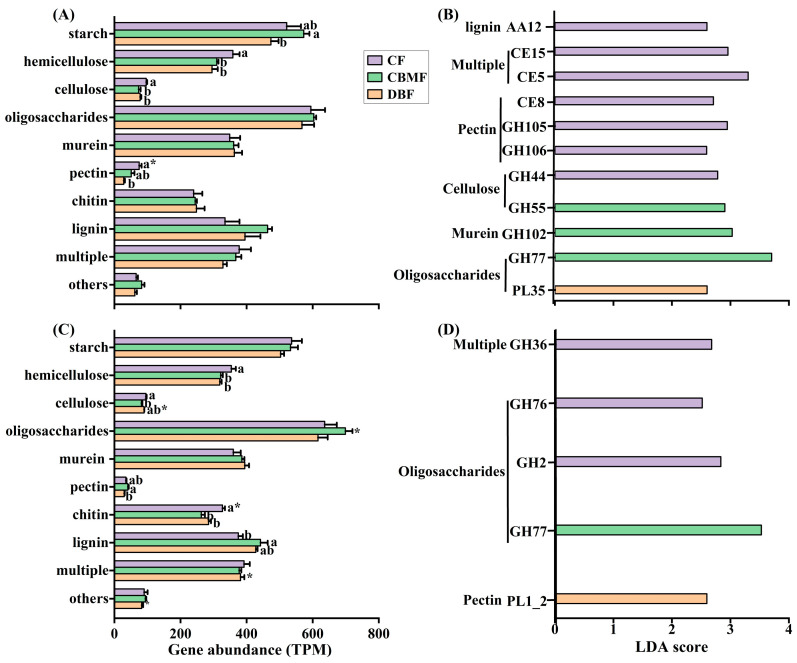
Changes in abundance of functional CAZyme genes encoding for carbon-degradation components among forest types in the 0–20 cm (**A**,**B**) and 20–40 cm (**C**,**D**) soil layer. Only genes that were significantly differentially abundant among forest types, with linear discriminant analysis (LDA) scores above 2.5 (*p* < 0.05), were shown. Different small letters indicate significant differences among forest types, and the * indicates significant differences between soil layers at a 0.05 significance level. CF, conifer forest; CBMF, conifer–broadleaf mixed forest; DBF, deciduous broadleaf forest.

The GHs (38.3–41.2%) and GTs (39.1–42.9%) were the most abundant CAZyme families and were significantly influenced by forest type, soil layer, and their interaction, as were PLs (Figure 5). In contrast, CBMs were affected only by soil layer, while CEs and AAs varied solely with forest type. Compared to DBF and CBMF, CF had a higher relative abundance of GHs (0–20 cm), CEs, and PLs, but a lower abundance of AAs. In the 20–40 cm layer, CBMF showed a higher abundance of GHs and a lower abundance of GTs (Figure 5A,B). While a considerable proportion of CAZyme genes involved in anabolic processes varied only with soil layer, genes related to the degradation of cellulose, chitin, and pectin were significantly affected by forest type and/or soil layer (Figure S1, Table S5). In the 0–20 cm layer, CBMF had a higher gene abundance for starch degradation than DBF, whereas CF showed higher gene abundance for cellulose and pectin degradation compared to both DBF and CBMF (Figure 6A). In the 20–40 cm layer, CBMF exhibited higher gene abundances for pectin and lignin degradation, while CF had higher gene abundances for chitin and pectin degradation (Figure 6C). LEfSe analysis further identified distinct CAZyme-family genes enriched across forests and depths (Figure 6B,D). CF was enriched in genes GH105, GH106, GH44, CE8, CE5, CE15, and AA12 in the 0–20 cm layer and genes GH2, GH36, and GH76 in the 20–40 cm layer. In contrast, CBMF showed enrichment of genes GH55, GH77, and GH102 (0–20 cm); DBF was enriched in gene PL35 (0–20 cm) and gene PL1_2 (20–40 cm).

### 3.5. Relationships Between Microbial Community, Carbon Function Genes, and Soil Properties

**Figure 7:**
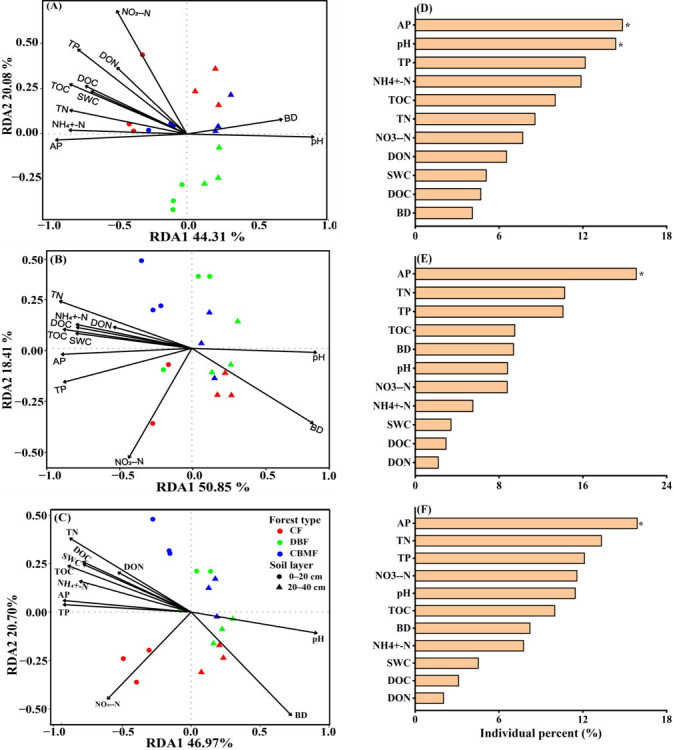
Distance-based redundancy analysis (db-RDA) of soil properties and microbial community (genus level, (**A**)), genes for carbon fixation (**B**), and CAZyme families (**C**) across forest types and soil layers. The relative contributions of each soil parameter to these variations are present (**D**–**F**). The * indicates significant differences between soil layers at a 0.05 significance level. CF, conifer forest; CBMF, conifer–broadleaf mixed forest; DBF, deciduous broadleaf forest. BD, bulk density; SWC, soil water content; TOC, total organic carbon; TN, total nitrogen; TP, total phosphorus; NH_4_^+^-N, ammonium; NO_3_^−^-N, nitrate; AP, available phosphorus; DOC, dissolved organic carbon; DON, dissolved organic nitrogen.

Pearson’s correlation analysis showed that soil pH and BD were negatively correlated with microbial diversity, specific genera (i.e., streptomyces, caulobacter, and trichoderma), and genes linked to CBMs, GHs, pectin-degradation, and C fixation (i.e., *IDH1*, *ALDO*, *metF*, *fumA*) (Figures S2 and S3). In contrast, they were positively correlated with genes for chitin degradation, *sdhA*, *korA/B*, and *accA/D*. Soil AP, TOC, and TN displayed generally opposite correlation patterns, while NO_3_^−^-N was negatively associated with genes for lignin degradation, *ackA*, *rpiA*, and *maeB*, but positively correlated to cellulose- and hemicellulose-degradation genes (Figure 7). The db-RDA analysis indicated that the first two axes explained 64.39% of the variance in microbial community structure, 69.26% in C-fixation genes, and 67.67% in CAZyme genes across forest types and soil layers (Figure 7A–C). Mantel tests further demonstrated that all measured soil properties except DON were significantly associated with microbial community composition and C-related functional genes (Table S6). Hierarchical partitioning identified that soil AP and pH were the key explanatory factors for microbial community variation, whereas AP was the key contributor to C-related functional genes (Figure 7D–F).

## 4. Discussion

Our results reveal distinct and often asynchronous responses of bacterial and fungal diversity to forest type and soil layer, in line with patterns observed in previous studies [10,12]. The higher bacterial diversity in the surface layer of CF relative to DBF and CBMF may be explained by the variations in soil nutrient availability. CF soils possessed elevated C and N levels, and bacterial diversity showed a positive correlation with NH_4_^+^-N, AP, and DON, supporting the established link between bacterial community and substrate quantity/quality [34]. Moreover, the consistent decrease in microbial diversity with soil depth across forests, widely observed in other soil ecosystems [35,36,37], likely reflects the reduction in labile C and nutrient resources in the deeper layer, which constrains microbial growth and diversity [38,39].

Shifts in microbial community composition further underscore the influence of forest type and soil layer. The predominance of Pseudomonadota and Actinomycetota in this study was consistent with reports from other subtropical forests [12], yet their relative abundances varied significantly among forest types. Notably, CF favored Actinomycetota, while CBMF and DBF had the higher proportions of Pseudomonadota and Acidobacteriota. These compositional shifts were attributed to vegetation-driven changes in soil properties shaping community assembly [3,12,15,40]. Previous studies have indicated that soil pH is a master regulator of microbial community, although bacteria and fungi have different pH-tolerance ranges [36,41,42,43]. In the present study, the variation in soil pH across forest types and its significant correlation with community structure act as a primary environmental filter, directly influencing microbial physiology and nutrient availability [36,43]. In parallel with pH, soil AP emerged as a key factor driving microbial community variation. This strong influence likely originates from the pervasive P-limitation situation in subtropical forest ecosystems [44], which tightly couples microbial niches’ specialization to P availability [45]. The increase in AP can reshape the microbial community by expanding ecological niche dimensions for P-competitive taxa [46]. Furthermore, AP may indirectly modulate microbial assembly by interacting with other soil factors. Since P input can enhance the availability of C and N substrates, creating synergistic shifts in the edaphic environment [47]. These proposed mechanisms are corroborated by strong correlations between dominant microbial taxa and NH_4_^+^ and DOC in our study.

Calvin cycle was identified as the predominant C-fixation pathway in this study, aligning with the findings in semi-arid grassland [48] and paddy [49] soils, but contrasting with the results from arid desert regions, where the reductive citrate cycle prevails [50]. This divergence may be attributed to the higher energy demand of the Calvin cycle, which could be less favored in nutrient-limited environments [51]. Consistently, the low abundance of the WL pathway in our study supports this interpretation, as the WL pathway is favored in anaerobic, oligotrophic habitats [50,52] and is unsuitable for the aerobic, nutrient-poor soils examined here. Furthermore, forest type and soil layer differentially influenced C-fixation pathway. While the Calvin cycle did not change with forest type, the relative abundance of other pathways (i.e., the reductive citrate cycle) varied significantly among forest types. Specifically, the reductive citrate cycle and WL pathway were more abundant in CBMF and CF, whereas the 3-hydroxypropionate bi-cycle and hydroxypropionate-hydroxybutylate cycle were enriched in DBF, particularly in surface soils. These variations were likely to be explained by the difference in genes encoding these pathways [53]. In the current study, significant variations were observed in the abundance of key functional genes, such as *sucD*, *IDH1*, *fumA*, and *ACO* (for the reductive citrate cycle) and *mcmA2* (for the 3-hydroxypropionate bi-cycle and hydroxypropionate-hydroxybutylate cycle). Intriguingly, the key Calvin cycle genes *cbbL* and *rpi* were distinctly enriched in CF and DBF, respectively, despite the overall stable abundance of this pathway. This indicates that the total pathway abundance can mask differential contributions for specific genes and suggests that the same metabolic function may be achieved through phylogenetic or genetic routes [50], highlighting functional redundancy and niche differentiation in C-fixation strategies within forest ecosystems.

For the genetic potential for C-decomposition, GHs and GTs were the most abundant CAZyme classes, with profiles differing by forest type and soil layer, consistent with patterns in temperate forests [9,25]. Furthermore, forest type exerted a significant influence on the relative abundances of CEs, AAs, and PLs. These CAZyme classes were closely linked to the degradation of diverse plant and microbial C compounds, as well as to soil C biosynthesis [10,28,54]. GHs and AAs are key drivers in the decomposition of polysaccharides (i.e., starch, hemicellulose, cellulose, and chitin) and lignin, respectively, while certain CEs participate in hemicellulose breakdown [7,10,55]. Interestingly, in this study, CF displayed a higher relative abundance of GHs and CEs, but lower AAs, especially in the surface layer. In contrast, CBMF showed an increased GH abundance in deeper soil layers. This differential distribution implies a potential shift in C-decomposition strategies: CF appears to be more oriented toward the processing of labile C substrates (i.e., pectin, cellulose), whereas CBMF may be more associated with the breakdown of recalcitrant C substrates (i.e., lignin). This interpretation aligns with established enzymatic functions and is supported by corresponding shifts in the abundance of specific gene families (i.e., CE8, GH105, GH44, and GH76) associated with the degradation of these compounds. This may reflect vegetation-specific inputs and the differing biochemical quality of litter.

Previous studies have consistently indicated that soil properties (i.e., pH, SWC) were closely associated with C-cycling [50,56,57]. Our results reveal that soil AP was the dominant factor explaining variation in both C-fixation and decomposition gene profiles, paralleling its strong influence on microbial community structure. This highlights the more central role of P in regulating microbial functional traits for C- cycling. The strong correlations between AP and the abundance of key C-fixation genes (i.e., *korA/B*, *accD*, *ppdK*, and *IDH1*) suggest that P may critically constrain microbial carbon assimilation, likely by limiting energy metabolism and nucleic acid synthesis in autotrophic microorganisms [45]. Similarly, for the decomposition process, AP was the primary driver shaping the composition of CAZyme genes. The correlation between AP and specific CAZyme families (i.e., pectin, chitin, and murein) further implies that P availability acts as a master variable, strategically modulating microbial investment in the synthesis of extracellular enzymes for C- processing [58,59]. This P-mediated regulation appears to follow a substrate-dependent investment strategy. Specifically, higher AP favored the genetic capacity for decomposing more labile carbon substrates like pectin, while correlating negatively with genes for degrading recalcitrant compounds such as chitin and lignin. This substrate-specific pattern was further nuanced by interactions with other soil variables: for instance, conditions favoring pectin decomposition (e.g., higher AP, SWC, and C/N) were typically unfavorable for chitin degradation. Additionally, nitrate availability promoted genes for hemicellulose and cellulose decomposition but suppressed those for lignin modification. Collectively, these results demonstrate that nutrient availability, with P playing a central role, interacts with the broader soil physicochemical matrix to shape distinct microbial metabolic strategies for the turnover of different C polymers.

## 5. Conclusions

Forest type, associated with soil layer, significantly influences soil C-cycling in subtropical forests by shaping distinct microbial communities and their functional potentials. Metagenomic analysis revealed that coniferous forest (CF) soils hosted a more diverse bacterial community with a greater capacity for complex carbon degradation. In contrast, mixed forests (CBMF) showed higher functional potential for starch and lignin breakdown. Primarily, soil available phosphorus (AP) and pH drove microbial community composition, whereas AP predominantly regulated the abundance and distribution of C-cycle genes. These findings underscore how forest type modulates belowground C processes through its selective effects on microbial structure and function, with soil properties acting as key mediators.

## Figures and Tables

**Table 1:** Soil physicochemical properties in different forest types.

Properties	DBF		CBMF		CF	
	0–20 cm	20–40 cm	0–20 cm	20–40 cm	0–20 cm	20–40 cm
BD (g cm^−3^)	0.85 ± 0.03 a	0.92 ± 0.02 a	0.71 ± 0.03 a	0.93 ± 0.02 b*	0.79 ± 0.04 a	1.05 ± 0.02 a*
pH	4.67 ± 0.02 a	4.83 ± 0.01 a*	4.53 ± 0.02 b	4.88 ± 0.04 a*	4.43 ± 0.02 c	4.88 ± 0.03 a*
SWC (%)	32.0 ± 0.32 a*	26.7 ± 0.92 a	33.5 ± 0.68 a*	30.0 ± 1.05 a	35.5 ± 2.1 a*	27.9 ± 1.8 a
TOC (g kg^−1^)	27.47 ± 1.27 b*	11.63 ± 0.42 b	44.55 ± 2.31 a*	19.07 ± 0.73 a	46.52 ± 2.53 a*	20.79 ± 1.48 a
TN (g kg^−1^)	2.28 ± 0.09 b*	0.96 ± 0.04 b	3.55 ± 0.13 a*	1.45 ± 0.03 a	3.23 ± 0.23 a*	1.30 ± 0.11 a
TP (g kg^−1^)	0.25 ± 0.01 b*	0.08 ± 0.01 b	0.38 ± 0.01 a*	0.26 ± 0.01 a	0.43 ± 0.03 a*	0.25 ± 0.01 a
NH_4_^+^-N (mg kg^−1^)	2.62 ± 0.09 b*	1.45 ± 0.25 a	2.68 ± 0.07 b*	1.31 ± 0.06 a	3.34 ± 0.27 a*	1.48 ± 0.2 a
NO_3_^−^-N (mg kg^−1^)	1.83 ± 0.10 c	1.58 ± 0.34 c	4.01 ± 0.51 b*	2.22 ± 0.33 b	7.08 ± 0.34 a	5.6 ± 0.52 a
AP (mg kg^−1^)	1.38 ± 0.25 b*	0.23 ± 0.09 a	1.4 ± 0.10 b*	0.21 ± 0.06 a	2.51 ± 0.14 a*	0.28 ± 0.06 a
DON (mg kg^−1^)	26.97 ± 1.36 a*	14.53 ± 1.57 a	29.37 ± 1.95 a*	21.42 ± 1.7 a	28.51 ± 1.51 a	25.9 ± 3.39 a
DOC (mg kg^−1^)	301.5 ± 4.0 b*	131.6 ± 15.3 b	368.4 ± 8.7 a*	232.9 ± 5.5 a	385.2 ± 22.7 a*	233.6 ± 30.0 a

Different small letters indicate significant differences among forest types, and the * indicates significant differences between soil layers in the same forest type at a 0.05 significance level. CF, conifer forest; CBMF, conifer–broadleaf mixed forest; DBF, deciduous broadleaf forest. BD, bulk density; SWC, soil water content; TOC, total organic carbon; TN, total nitrogen; TP, total phosphorus; NH_4_^+^-N, ammonium; NO_3_^−^-N, nitrate; AP, available phosphorus; DOC, dissolved organic carbon; DON, dissolved organic nitrogen.

## Data Availability

The original contributions presented in this study are included in the article/Supplementary Materials. Further inquiries can be directed to the corresponding author.

## Supplementary Materials

The following supporting information can be downloaded at https://www.mdpi.com/article/10.3390/microorganisms14010220/s1, Figure S1: Relative abundance of CAZyme gene families involved in carbon degradation in different forest types. CF, conifer forest; CBMF, conifer–broadleaf mixed forest; DBF, deciduous broadleaf forest.; Figure S2: Pearson correlations between soil properties and main microbial genus (A), CAZyme genes (B). * and ** represent at 0.05 and 0.01 significance levels, respectively. AAs, auxiliary activities; GHs, glycoside hydrolases; GTs, glycosyl transferases; PLs, polysaccharide lyases; CEs, carbohydrate esterases; CBMs, carbohydrate-binding modules. BD, bulk density; SWC, soil water content; TOC, total organic carbon; TN, total nitrogen; TP, total phosphorus; NH_4_^+^-N, ammonium; NO_3_^-^-N, nitrate; AP, available phosphorus; DOC, dissolved organic carbon; DON, dissolved organic nitrogen.; Figure S3: Pearson correlations between soil properties and carbon fixation genes. * and ** represent at 0.05 and 0.01 significance levels, respectively. BD, bulk density; SWC, soil water content; TOC, total organic carbon; TN, total nitrogen; TP, total phosphorus; NH_4_^+^-N, ammonium; NO_3_^-^-N, nitrate; AP, available phosphorus; DOC, dissolved organic carbon; DON, dissolved organic nitrogen.; Table S1: Basic information of metagenomic sequencing; Table S2: Relative abundance of bacteria, fungi, and archaea in metagenomic reads; Table S3: Differences in community composition of bacteria and fungi and the effects of forest type (FT) and soil layer (SL) to them at phyla level; Table S4: Information of microbial functional genes involved in carbon fixation identified in this study; Table S5: Effects of forest type (FT) and soil layer (SL) on carbon fixation pathways and carbon degradation in CAZyme gene families; Table S6: Significance effect of soil properties to microbial communities, carbon fixation, and CAZyme genes were examined using the Mantel Test (permutation = 999), and the significance was analyzed by the R^2^ and *p* value.
