# Supplementary material for: Forest Type Shapes Soil Microbial Carbon Metabolism: A Metagenomic Study of Subtropical Forests on Lushan Mountain
Source: Microorganisms. 2026 Jan 17;14(1):220. doi: 10.3390/microorganisms14010220 (PMC12844230; doi:10.3390/microorganisms14010220)
Supplement: Supplementary file 1 [file microorganisms-14-00220-s001.zip › microorganisms-4058037-supplementary.pdf]

Supplementary Materials

Forest type shapes soil microbial carbon metabolism: a metagenomic study of subtropical forests on Lushan Mountain

Dan Xi [1*], Feifei Zhu [2], Zhaochen Zhang [1], Saixia Zhou[1], Jiaxin Zhang[1]

[1] Jiangxi Provincial Key Laboratory of Carbon Neutrality and Ecosystem Carbon Sink, Lushan Botanical Garden, Jiangxi Province and Chinese Academy of Sciences, Jiujiang, 332900, China

[2] Institute of Applied Ecology, Chinese Academy of Sciences, Shenyang, 110016, China

* Correspondence: xidan@lsbg.cn

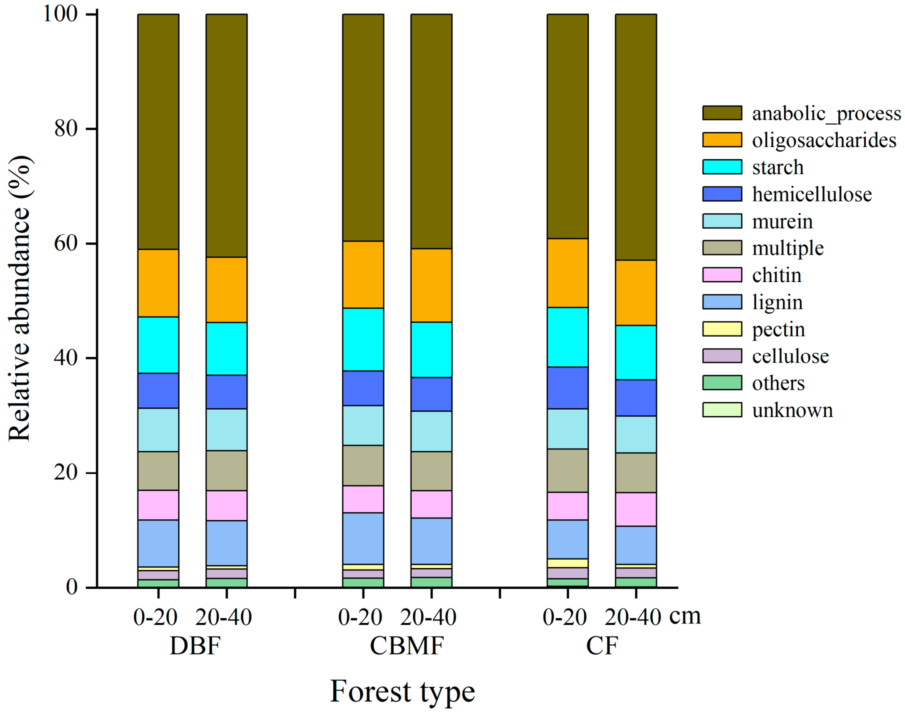

Figure S1 Relative abundance of CAZyme gene families involved in carbon degradation in different forest types. CF, conifer forest; CBMF, conifer-broadleaf mixed forest; DBF, deciduous broadleaf forest.

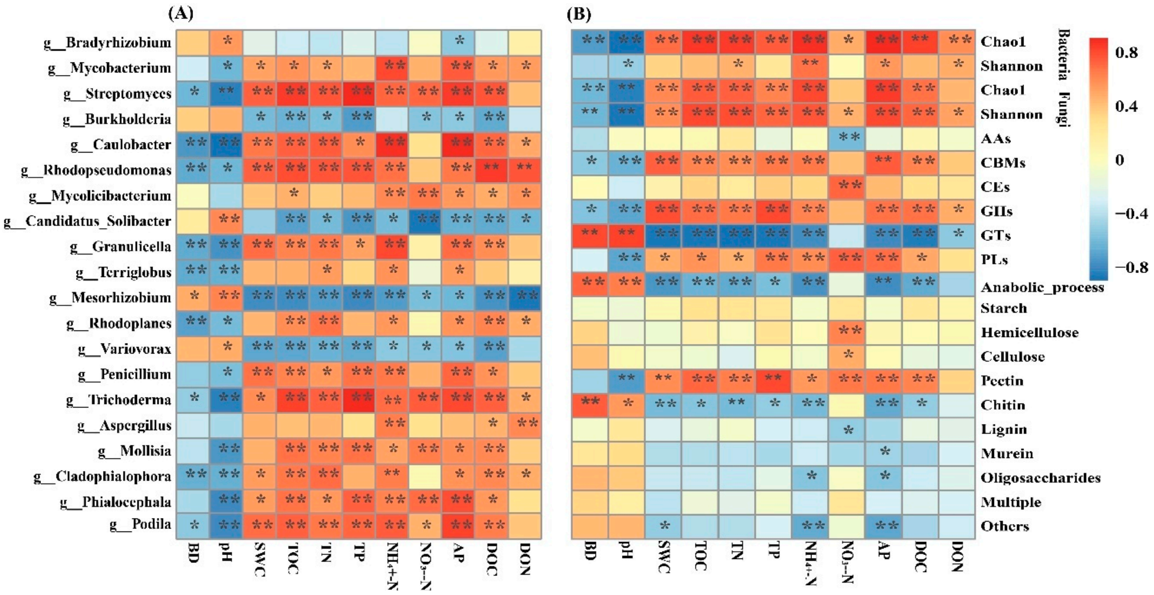

Figure S2 Pearson correlations between soil properties and main microbial genus (A), CAZyme genes (B). * and ** represent significance at 0.05 and 0.01 levels, respectively. BD, bulk density; SWC, soil water content; TOC, total organic carbon; TN, total nitrogen; TP, total phosphorus; NH_4^+-N, ammonium; NO_3^--N, nitrate; AP, available phosphorus; DOC, dissolved organic carbon; DON, dissolved organic nitrogen.

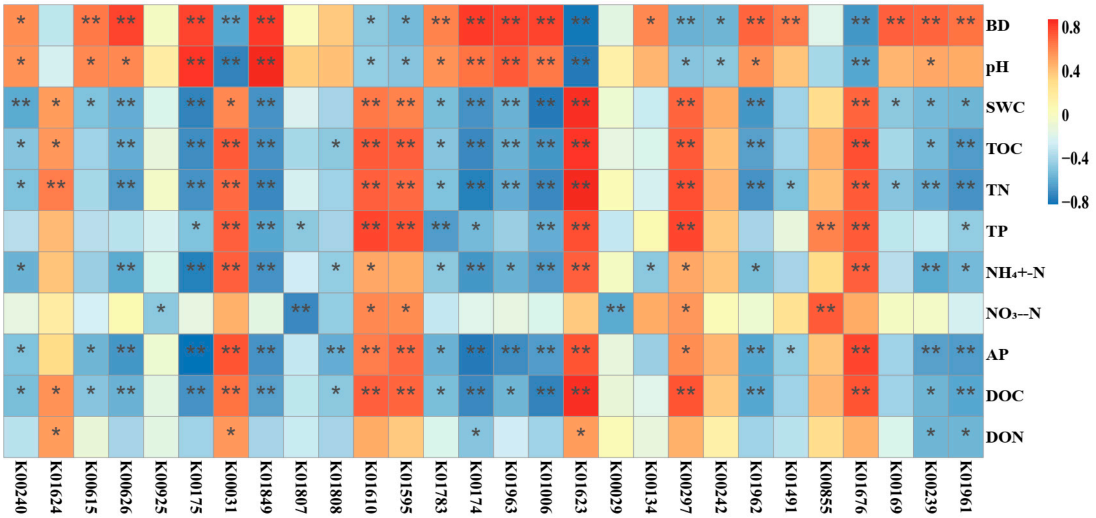

Figure S3 Pearson correlations between soil properties and carbon fixation genes. * and ** represent significance at 0.05 and 0.01 levels, respectively. BD, bulk density; SWC, soil water content; TOC, total organic carbon; TN, total nitrogen; TP, total phosphorus; NH_4^+-N, ammonium; NO_3^--N, nitrate; AP, available phosphorus; DOC, dissolved organic carbon; DON, dissolved organic nitrogen.

Table S1 Basic information of metagenomic sequencing.

Samples	Soil layer (cm)	RawReads	CleanReads	Clean%[a]	Contigs	N50[b]	GC (%)[c]	ORFs[d]
CF_1	0-20	33,576,993	32,400,090	96.49491	305,524	706	61.25	250828
CF_2	0-20	34,652,720	33,336,844	96.20268	269,528	693	60.49	219904
CF_3	0-20	34,253,032	33,177,659	96.8605	324,433	766	60.23	289695
CF_4	20-40	35,775,066	34,277,073	95.81275	456,643	765	60.23	399429
CF_5	20-40	33,770,997	32,571,977	96.44956	423,845	770	59.86	370993
CF_6	20-40	32,087,402	30,891,418	96.27273	412,261	793	60.04	375466
DBF_1	0-20	33,526,209	32,176,245	95.97341	248,000	691	60.3	198464
DBF_2	0-20	33,657,880	32,511,853	96.59507	304,173	729	59.62	253571
DBF_3	0-20	34,034,087	32,644,848	95.9181	267,180	706	59.51	216952
DBF_4	20-40	33,062,153	32,054,380	96.95188	341,946	746	60.01	293245
DBF_5	20-40	33,999,166	32,832,848	96.56957	454,283	778	59.93	403640
DBF_6	20-40	33,308,411	32,184,810	96.62667	410,407	739	59.67	347713
CBMF_1	0-20	34,097,408	32,583,518	95.5601	293,514	729	59.98	250766
CBMF_2	0-20	33,823,996	32,661,471	96.56302	282,453	700	59.87	227126
CBMF_3	0-20	33,711,397	32,289,629	95.78253	305,184	735	60.31	261485
CBMF_4	20-40	32,180,781	30,730,143	95.49222	390,586	738	59.94	335713
CBMF_5	20-40	34,324,933	32,996,619	96.13018	407,752	728	59.45	340009
CBMF_6	20-40	32,405,335	31,004,518	95.6772	322,044	699	59.85	262463

[a] The percentage of clean read in its corresponding raw read.

[b] N50 represents the length of the contig overlapping the midpoint of the length-order concatenation of contigs.

[C] GC represents the percentage of bases G and C to total bases (G+C+ A,+ T) in contings.

[D] ORFs Open reading frames.

CF, conifer forest; CBMF, conifer-broadleaf mixed forest; DBF, deciduous broadleaf forest.

Table S2 Relative abundance of bacteria, fungi, and archaea in metagenomic reads.

Forest type	Soil layer	Relative abundance (%)		
	(cm)	bacteria	fungi	archaea
DBF	0-20	99.6 ±0.05	0.25±0.04	0.18±0.01
	20-40	99.7±0.02	0.13±0.01	0.20±0.02
CBMF	0-20	99.7±0.07	0.22±0.07	0.11±0.01
	20-40	99.7±0.04	0.09±0.02	0.20±0.03
PF	0-20	99.6±0.03	0.30±0.01	0.14±0.02
	20-40	99.6±0.06	0.12±0.02	0.32±0.04

CF, conifer forest; CBMF, conifer-broadleaf mixed forest; DBF, deciduous broadleaf forest.

Table S3 Differences in community composition of bacterial and fungi and the effects of forest type (FT) and soil layer (SL) to them at phyla level.

	Phylum	DBF		CBMF		CF		FT	SL	FT×SL
		0-20 cm	20-40 cm	0-20 cm	20-40 cm	0-20 cm	20-40 cm	*P*	*P*	*P*
Baceria	Pseudomonadota	55.9± 0.22a	53.3±1.2a	56.9±1.5a	54.3±0.71a	52.2±0.38b	53.5±1.1a	**0.049**	0.129	0.113
	Actinomycetota	12.2±0.28b	10.7±0.50ab	13.4±0.56b*	9.7±0.59b	19.1±0.84a*	12.6±0.90a	**<0.001**	**<0.001**	**0.008**
	Acidobacteriota	1.01±0.04a	1.05±0.04a	0.93±0.07a	1.02±0.10a	0.84±0.02b	0.80±0.06b	**0.012**	0.503	0.544
	Planctomycetota	0.72±0.04a	0.74±0.01a	0.46±0.03b	0.68±0.04ab*	0.43±0.02b	0.58±0.03b*	**<0.001**	**<0.001**	**0.037**
	Myxococcota	0.53±0.03a	0.53±0.01a	0.38±0.007b	0.48±0.01b*	0.35±0.03b	0.47±0.02b*	**<0.001**	**0.001**	**0.031**
	Bacillota	0.07±0.001ab	0.10±0.007a*	0.08±0.007a	0.11±0.008a	0.06±0.004b	0.09±0.004a*	**0.029**	**<0.001**	0.720
	Bacteroidota	0.12±0.01ab*	0.06±0.01a	0.08±0.008b*	0.04±0.004a	0.15±0.02a*	0.07±0.01a	**0.004**	**<0.001**	0.212
	Gemmatimonadota	0.04±0.002a	0.04±0.005a	0.04±0.003a	0.06±0.01a*	0.03±0.003a	0.05±0.002a*	**0.052**	**0.016**	0.167
	Chloroflexota	0.03±0.001a	0.07±0.006a*	0.02±0.001a	0.05±0.004a*	0.02±0.003a	0.07±0.01a*	0.111	**<0.001**	0.304
	Verrucomicrobiota	0.05±0.005a	0.05±0.002a	0.05±0.002a	0.05±0.005a	0.06±0.003a	0.04±0.003a*	0.892	**0.020**	0.196
	Nitrospirota	0.03±0.002a	0.04±0.003b	0.02±0.008b	0.06±0.002a*	0.01±0.001c	0.04±0.005b*	**0.001**	**0.001**	**0.004**
	Others	0.02±0.001a	0.01±0.002a	0.02±0.001a	0.02±0.004a	0.02±0.002aa	0.02±0.001a	0.686	0.965	0.166
	Unclassified	29.2±0.09a	33.3±1.2*	27.9±1.0a	33.4±0.68*	26.7±0.61a	31.6±1.1a*	0.077	**<0.001**	0.625
Fungi	Ascomycota	48.8±8.4a	46.7±3.6ab	48.8±4.2a	41.7±3.8b	63.0±6.1a	53.8±1.3a	**0.050**	0.162	0.775
	Basidiomycota	17.6±9.0a	20.2±8.5a	18.7±5.0a	24.4±6.9a	11.2±1.1a	18.3±1.4a*	0.556	0.331	0.936
	Mucoromycota	17.6±8.7a	11.1±10.4a	13.7±11.1a	10.2±5.2a	8.7±7.3a	3.8±1.4a	0.602	0.463	0.983
	Unclassified	16.0±1.4a	22.0±2.8a*	18.7±2.0a	23.7±1.2a*	17.0±1.4a	24.1±1.4a*	0.460	**0.001**	0.841

Notes: Different small letters indicate significant difference among different forest types, and the * indicates significant difference between soil layers at 0.05 level. CF, conifer forest; CBMF, conifer-broadleaf mixed forest; DBF, deciduous broadleaf forest.

Table S4 Information of microbial functional genes involved in carbon fixation identified in this study.

Modules	Pathways	KO number	Gene name	Gene function
M00165	Reductive pentose phosphate cycle (Calvin cycle)	K00134	GAPDH, gapA	glyceraldehyde 3-phosphate dehydrogenase (phosphorylating) [EC:1.2.1.12]
		K00150	gap2, gapB	glyceraldehyde-3-phosphate dehydrogenase (NAD(P)+) (phosphorylating) [EC:1.2.1.59]
		K00615	E2.2.1.1, tktA, tktB	transketolase [EC:2.2.1.1]
		K00855	PRK, prkB	phosphoribulokinase [EC:2.7.1.19]
		K00927	PGK, pgk	phosphoglycerate kinase [EC:2.7.2.3]
		K01601	rbcL, cbbL	ribulose-bisphosphate carboxylase large chain [EC:4.1.1.39]
		K01602	rbcS, cbbS	ribulose-bisphosphate carboxylase small chain [EC:4.1.1.39]
		K01623	ALDO	fructose-bisphosphate aldolase, class I [EC:4.1.2.13]
		K01624	FBA, fbaA	fructose-bisphosphate aldolase, class II [EC:4.1.2.13]
		K01783	rpe, RPE	ribulose-phosphate 3-epimerase [EC:5.1.3.1]
		K01803	TPI, tpiA	triosephosphate isomerase (TIM) [EC:5.3.1.1]
		K01807	rpiA	ribose 5-phosphate isomerase A [EC:5.3.1.6]
		K01808	rpiB	ribose 5-phosphate isomerase B [EC:5.3.1.6]
		K02446	glpX	fructose-1,6-bisphosphatase II [EC:3.1.3.11]
		K03841	FBP, fbp	fructose-1,6-bisphosphatase I [EC:3.1.3.11]
		K11532	glpX-SEBP	fructose-1,6-bisphosphatase II / sedoheptulose-1,7-bisphosphatase [EC:3.1.3.11 3.1.3.37]
M00168	CAM (Crassulacean acid metabolism), dark	K00024	mdh	malate dehydrogenase [EC:1.1.1.37]
		K01595	ppc	phosphoenolpyruvate carboxylase [EC:4.1.1.31]
M00169	CAM (Crassulacean acid metabolism), light	K00029	maeB	malate dehydrogenase (oxaloacetate-decarboxylating)(NADP+) [EC:1.1.1.40]
		K01006	ppdK	pyruvate, orthophosphate dikinase [EC:2.7.9.1]
M00170	C4-dicarboxylic acid cycle, phosphoenolpyruvate carboxykinase type	K01595	ppc	phosphoenolpyruvate carboxylase [EC:4.1.1.31]
		K01610	pckA	phosphoenolpyruvate carboxykinase (ATP) [EC:4.1.1.49]
M00171	C4-dicarboxylic acid cycle, NAD - malic enzyme type	K01006	ppdK	pyruvate, orthophosphate dikinase [EC:2.7.9.1]
M00172	C4-dicarboxylic acid cycle, NADP - malic enzyme type	K00029	maeB	malate dehydrogenase (oxaloacetate-decarboxylating)(NADP+) [EC:1.1.1.40]
		K01006	ppdK	pyruvate, orthophosphate dikinase [EC:2.7.9.1]
		K01595	ppc	phosphoenolpyruvate carboxylase [EC:4.1.1.31]
		K00024	mdh	malate dehydrogenase [EC:1.1.1.37]
M00173	Reductive citrate cycle (Arnon-Buchanan cycle)	K00024	mdh	malate dehydrogenase [EC:1.1.1.37]
		K00031	IDH1, IDH2	isocitrate dehydrogenase [EC:1.1.1.42]
		K00169	porA	pyruvate ferredoxin oxidoreductase alpha subunit [EC:1.2.7.1]
		K00170	porB	pyruvate ferredoxin oxidoreductase beta subunit [EC:1.2.7.1]
		K00174	korA, oorA, oforA	2-oxoglutarate/2-oxoacid ferredoxin oxidoreductase subunit alpha [EC:1.2.7.3 1.2.7.11]
		K00175	korB, oorB, oforB	2-oxoglutarate/2-oxoacid ferredoxin oxidoreductase subunit beta [EC:1.2.7.3 1.2.7.11]
		K00239	sdhA, frdA	succinate dehydrogenase flavoprotein subunit [EC:1.3.5.1]

		K00240	sdhB, frdB	succinate dehydrogenase iron-sulfur subunit [EC:1.3.5.1]
		K00241	sdhC, frdC	succinate dehydrogenase cytochrome b subunit
		K00242	sdhD, frdD	succinate dehydrogenase membrane anchor subunit
		K01006	ppdK	pyruvate, orthophosphate dikinase [EC:2.7.9.1]
		K01007	pps, ppsA	pyruvate, water dikinase [EC:2.7.9.2]
		K01595	ppc	phosphoenolpyruvate carboxylase [EC:4.1.1.31]
		K01676	E4.2.1.2A, fumA, fumB	fumarate hydratase, class I [EC:4.2.1.2]
		K01679	E4.2.1.2B, fumC, FH	fumarate hydratase, class II [EC:4.2.1.2]
		K01681	ACO, acnA	aconitate hydratase [EC:4.2.1.3]
		K01902	sucD	succinyl-CoA synthetase alpha subunit [EC:6.2.1.5]
		K01903	sucC	succinyl-CoA synthetase beta subunit [EC:6.2.1.5]
		K03737	por, nifJ	pyruvate-ferredoxin/flavodoxin oxidoreductase [EC:1.2.7.1 1.2.7.-]
M00374	Dicarboxylate-hydroxybutyrate cycle	K00024	mdh	malate dehydrogenase [EC:1.1.1.37]
		K00169	porA	pyruvate ferredoxin oxidoreductase alpha subunit [EC:1.2.7.1]
		K00170	porB	pyruvate ferredoxin oxidoreductase beta subunit [EC:1.2.7.1]
		K00239	sdhA, frdA	succinate dehydrogenase flavoprotein subunit [EC:1.3.5.1]
		K00240	sdhB, frdB	succinate dehydrogenase iron-sulfur subunit [EC:1.3.5.1]
		K00241	sdhC, frdC	succinate dehydrogenase cytochrome b subunit
		K00626	ACAT, atoB	acetyl-CoA C-acetyltransferase [EC:2.3.1.9]
		K01007	pps, ppsA	pyruvate, water dikinase [EC:2.7.9.2]
		K01595	ppc	phosphoenolpyruvate carboxylase [EC:4.1.1.31]
		K01902	sucD	succinyl-CoA synthetase alpha subunit [EC:6.2.1.5]
		K01903	sucC	succinyl-CoA synthetase beta subunit [EC:6.2.1.5]
		K14534	abfD	4-hydroxybutyryl-CoA dehydratase / vinylacetyl-CoA-Delta-isomerase [EC:4.2.1.120 5.3.3.3]
M00375	Hydroxypropionate-hydroxybutylate cycle	K00626	ACAT, atoB	acetyl-CoA C-acetyltransferase [EC:2.3.1.9]
		K01848	E5.4.99.2A, mcmA1	methylmalonyl-CoA mutase, N-terminal domain [EC:5.4.99.2]
		K01849	E5.4.99.2B, mcmA2	methylmalonyl-CoA mutase, C-terminal domain [EC:5.4.99.2]
		K05606	MCEE, epi	methylmalonyl-CoA/ethylmalonyl-CoA epimerase [EC:5.1.99.1]
		K14534	abfD	4-hydroxybutyryl-CoA dehydratase / vinylacetyl-CoA-Delta-isomerase [EC:4.2.1.120 5.3.3.3]
M00376	3-Hydroxypropionate bi-cycle	K00239	sdhA, frdA	succinate dehydrogenase flavoprotein subunit [EC:1.3.5.1]
		K00240	sdhB, frdB	succinate dehydrogenase iron-sulfur subunit [EC:1.3.5.1]
		K00241	sdhC, frdC	succinate dehydrogenase cytochrome b subunit
		K01679	E4.2.1.2B, fumC	fumarate hydratase, class II [EC:4.2.1.2]
		K01847	MUT	methylmalonyl-CoA mutase [EC:5.4.99.2]
		K01848	E5.4.99.2A, mcmA1	methylmalonyl-CoA mutase, N-terminal domain [EC:5.4.99.2]
		K01849	E5.4.99.2B, mcmA2	methylmalonyl-CoA mutase, C-terminal domain [EC:5.4.99.2]
		K01961	accC	acetyl-CoA carboxylase, biotin carboxylase subunit [EC:6.4.1.2 6.3.4.14]
		K01962	accA	acetyl-CoA carboxylase carboxyl transferase subunit alpha [EC:6.4.1.2 2.1.3.15]
		K01963	accD	acetyl-CoA carboxylase carboxyl transferase subunit beta [EC:6.4.1.2 2.1.3.15]
		K02160	accB, bccP	acetyl-CoA carboxylase biotin carboxyl carrier protein
		K05606	MCEE, epi	methylmalonyl-CoA/ethylmalonyl-CoA epimerase [EC:5.1.99.1]
		K09709	meh	3-methylfumaryl-CoA hydratase [EC:4.2.1.153]

		K14449	mch, mcd	2-methylfumaryl-CoA hydratase [EC:4.2.1.148]
M00377	Reductive acetyl-CoA pathway (Wood-Ljungdahl pathway)	K00196	cooF	anaerobic carbon-monoxide dehydrogenase iron sulfur subunit
		K00297	metF, MTHFR	methylenetetrahydrofolate reductase (NADH) [EC:1.5.1.54]
		K01491	folD	methylenetetrahydrofolate dehydrogenase (NADP+) / methenyltetrahydrofolate cyclohydrolase [EC:1.5.1.5 3.5.4.9]
		K01500	fchA	methenyltetrahydrofolate cyclohydrolase [EC:3.5.4.9]
		K15022	fdhB	formate dehydrogenase (NADP+) beta subunit [EC:1.17.1.10]
M00579	Phosphate acetyltransferase-acetate kinase pathway, acetyl-CoA => acetate	K00625	pta	phosphate acetyltransferase [EC:2.3.1.8]
		K00925	ackA	acetate kinase [EC:2.7.2.1]
		K13788	pta	phosphate acetyltransferase [EC:2.3.1.8]
M00620	Incomplete reductive citrate cycle, acetyl-CoA => oxoglutarate	K00024	mdh	malate dehydrogenase [EC:1.1.1.37]
		K00169	porA	pyruvate ferredoxin oxidoreductase alpha subunit [EC:1.2.7.1]
		K00170	porB	pyruvate ferredoxin oxidoreductase beta subunit [EC:1.2.7.1]
		K00174	korA, oorA, oforA	2-oxoglutarate/2-oxoacid ferredoxin oxidoreductase subunit alpha [EC:1.2.7.3 1.2.7.11]
		K00175	korB, oorB, oforB	2-oxoglutarate/2-oxoacid ferredoxin oxidoreductase subunit beta [EC:1.2.7.3 1.2.7.11]
		K01902	sucD	succinyl-CoA synthetase alpha subunit [EC:6.2.1.5]
		K01903	sucC	succinyl-CoA synthetase beta subunit [EC:6.2.1.5]

Table S5 Effects of forest type (FT) and soil layer (SL) on carbon fixation pathways and carbon degradation in CAZyme gene families.

Parameters		**FT**		SL		FT × SL	
		F	*P*	F	*P*	F	*P*
Carbon fixation pathways	M00165 Reductive pentose phosphate cycle (Calvin cycle)	1.680	0.227	7.879	**0.016**	0.850	0.451
	M00168 CAM (Crassulacean acid metabolism), dark	2.105	0.165	10.392	**0.007**	1.669	0.229
	M00169 CAM (Crassulacean acid metabolism), light	7.731	**0.007**	0.763	0.400	2.969	0.090
	M00170 C4-dicarboxylic acid cycle, NAD - malic enzyme type	6.636	**0.011**	11.940	**0.005**	0.518	0.608
	M00171 C4-dicarboxylic acid cycle, NADP - malic enzyme type	2.058	0.170	26.386	**<0.001**	3.896	0.050
	M00172 C4-dicarboxylic acid cycle, phosphoenolpyruvate carboxykinase type	6.475	**0.012**	1.190	0.297	1.639	0.235
	M00173 Reductive citrate cycle (Arnon-Buchanan cycle)	1.616	0.239	5.905	**0.032**	5.059	**0.026**
	M00374 Dicarboxylate-hydroxybutyrate cycle	0.597	0.566	6.461	**0.026**	0.557	0.587
	M00375 Hydroxypropionate-hydroxybutylate cycle	5.706	**0.018**	27.125	**<0.001**	0.719	0.507
	M00376 3-Hydroxypropionate bi-cycle	7.270	**0.009**	16.490	**0.002**	3.087	0.083
	M00377 Reductive acetyl-CoA pathway (Wood-Ljungdahl pathway)	8.835	**0.004**	3.809	0.075	1.044	0.382
	M00579 Phosphate acetyltransferase-acetate kinase pathway	3.459	0.065	3.021	0.108	0.517	0.609
	M00620 Incomplete reductive citrate cycle	0.057	0.945	29.148	**<0.001**	1.354	0.295
CAZy gene families	Anabolic process	0.077	0.927	18.470	**0.001**	1.066	0.375
	Cellulose	14.109	**0.001**	5.371	**0.039**	1.902	0.192
	Chitin	1.714	0.221	13.057	**0.004**	2.455	0.128
	Hemicellulose	8.293	**0.005**	0.979	0.342	0.611	0.559
	Lignin	5.868	**0.017**	0.530	0.481	0.684	0.523
	Multiple	1.264	0.318	2.826	0.119	0.771	0.484
	Murein	0.789	0.476	1.839	0.200	0.133	0.877
	Oligosaccharides	1.812	0.205	5.858	**0.032**	0.438	0.655
	Pectin	11.885	**0.001**	13.959	**0.003**	7.951	**0.006**
	Starch	2.999	0.088	0.005	0.946	0.940	0.417
	Others	2.928	0.092	11.787	**0.005**	0.505	0.616

Table S6 Significance effect of soil properties to microbial communities, carbon fixation, and CAZyme genes were examined using the Mantel Test (permutation = 999), and the significance was analyzed by the R^2 and *P* value.

Variable	Microbial communities		Carbon fixation genes		CAZyme genes	
	R^2	*P* value	R^2	*P* value	R^2	*P* value
BD	0.089	0.007	0.229	0.002	0.157	0.006
pH	0.489	0.002	0.406	0.002	0.464	0.002
SWC	0.077	0.009	0.167	0.002	0.129	0.002
TOC	0.324	0.002	0.307	0.002	0.261	0.002
TN	0.354	0.002	0.406	0.002	0.341	0.002
TP	0.250	0.002	0.394	0.002	0.384	0.002
NH_4^+-N	0.276	0.002	0.215	0.006	0.187	0.002
NO_3^--N	0.080	0.011	0.042	0.024	0.082	0.009
AP	0.496	0.002	0.475	0.002	0.447	0.002
DON	0.144	0.002	0.107	0.009	0.078	0.022
DOC	0.009	0.162	0.000	0.539	0.013	0.832

BD, bulk density; SWC, soil water content; TOC, total organic carbon; TN, total nitrogen; TP, total phosphorus; NH_4^+-N, ammonium; NO_3^--N, nitrate; AP, available phosphorus; DOC, dissolved organic carbon; DON, dissolved organic nitrogen.
